# Dissecting Immunotherapy Strategies for Small Cell Lung Cancer: Antibodies, Ionizing Radiation and CAR-T

**DOI:** 10.3390/ijms232112728

**Published:** 2022-10-22

**Authors:** Giorgia Guaitoli, Giovanni Neri, Eleonora Cabitza, Salvatore Natalizio, Luciana Mastrodomenico, Sabrina Talerico, Lucia Trudu, Chiara Lauro, Chiara Chiavelli, Maria Cristina Baschieri, Alessio Bruni, Massimo Dominici, Federica Bertolini

**Affiliations:** 1 PhD Program Clinical and Experimental Medicine, University of Modena and Reggio Emilia, 41125 Modena, Italy; 2 Laboratory of Cellular Therapy, Division of Oncology, Department of Medical and Surgical Sciences for Children & Adults, University of Modena and Reggio Emilia, 41124 Modena, Italy; 3 Division of Oncology, Department of Oncology and Hematology, Modena University Hospital, 41124 Modena, Italy; 4 Radiotherapy Unit, Department of Oncology and Hematology, Modena University Hospital, 41124 Modena, Italy

**Keywords:** small cell lung cancer, immune checkpoint inhibitors, PD-(L)1, biomarkers, drug conjugates

## Abstract

Small cell lung cancer (SCLC) is a highly aggressive malignancy that accounts for about 14% of all lung cancers. Platinum-based chemotherapy has been the only available treatment for a long time, until the introduction of immune checkpoint inhibitors (ICIs) recently changed first-line standard of care and shed light on the pivotal role of the immune system. Despite improved survival in a subset of patients, a lot of them still do not benefit from first-line chemo-immunotherapy, and several studies are investigating whether different combination strategies (with both systemic and local treatments, such as radiotherapy) may improve patient outcomes. Moreover, research of biomarkers that may be used to predict patients’ outcomes is ongoing. In addition to ICIs, immunotherapy offers other different strategies, including naked monoclonal antibodies targeting tumor associated antigens, conjugated antibody, bispecific antibodies and cellular therapies. In this review, we summarize the main evidence available about the use of immunotherapy in SCLC, the rationale behind combination strategies and the studies that are currently ongoing in this setting, in order to give the reader a clear and complete view of this rapidly expanding topic.

## 1. Introduction

Small cell lung cancer (SCLC) is a highly malignant neuroendocrine tumor, and represents the most aggressive type of lung cancer. Lung cancer represents approximately 11% of new cancer cases worldwide, and remains the leading cause of cancer-related death. SCLC accounts for about 14% of all lung cancers (82% is represented by non-small cell lung cancer [NSCLC], it is most prevalent in men, and smoking is the major risk factor for its development) [1].

Given the lack of specific symptoms in the early stages of disease and its tendency to early dissemination, the vast majority of patients are diagnosed with extensive stage (ES-SCLC) disease.

Despite the fact that SCLC demonstrated good response to first line standard therapy, with objective response rates (ORR) ≥50%, this form of lung cancer is known to be aggressive. Indeed, disease invariably progresses and the 5-year general survival rate is only 7% [1].

With a median progression-free survival (PFS) and overall survival (OS) of about 6 and 10 months, respectively, platinum plus etoposide doublet (EP) has for a long time been the preferred first-line option for ES-SCLC, and very little progress was made in the treatment of SCLC prior to the era of immune checkpoint inhibitors (ICIs) [2].

The assumption that immune modulation may have a role in the treatment of SCLC is supported by the evidence that the disease has classically been associated with immune-mediated paraneoplastic processes [3,4].

SCLC is also usually characterized by a high tumor mutational burden (TMB) [5,6,7] due to its strong association with smoking [8], and SCLC samples are invariably characterized by loss of functional tumor protein p53 (TP53) and Retinoblastoma 1 (Rb) [5]. Moreover, alterations such as *MYC* amplification or *PTEN* mutations, and mutations/amplification of SOX family genes, were described [6,7]. Overall, these alterations can lead to rapid proliferation, replicative stress and may promote the generation of tumor associated antigens (TAA).

All of these features are encouraging premises for the role of immunotherapy in the treatment of SCLC, and some improvements have been made in the last years since the introduction in clinical practice of agents targeting programmed cell death protein-1 (PD-1) or programmed death-ligand 1 (PD-L1). Despite this, many issues still persist and many obstacles need to be overcome to consistently improve patients’ prognosis.

In this review article, we will summarize the main evidence available about the use of immunotherapy in this setting, the rationale behind the combination with other systemic and local treatments, and the ongoing studies. We will also report evidence about possible biomarkers that may be used to predict patients’ outcomes.

## 2. “First Steps” of Immunotherapy in ES-SCLC

As known, although SCLC is a particularly chemo-sensitive disease, long-lasting treatment options are lacking [9]. Indeed, after platinum-based first line treatment, most patients experience poor prognosis due to early relapse. For years, despite poor median OS (mOS), topotecan has been the only second-line treatment option [10]. The decades-long search for a viable treatment option in relapsed SCLC led to immunotherapy when the anti-PD-1 monoclonal antibody nivolumab (alone or in combination with ipilimumab) demonstrated activity in relapse/refractory SCLC in the phase 1/2 trial CheckMate-032, with benefit consistent among different subgroups except patients with ECOG performance status 1 [11].

After these results, many ICIs have been investigated, at first in pretreated patients, in SCLC (Table 1).

Even pembrolizumab (anti-PD-1 agent) showed durable activity in a subset of patients with relapsed or metastatic SCLC treated with at least two lines of therapy. In the Keynote-028 (phase 1) and in Keynote-158 (phase 2) trials, patients received pembrolizumab for up to 2 years (10 mg/kg every 2 weeks or 200 mg every 3 weeks, respectively). The pooled analysis of these two studies reported two complete and 14 partial responses with an ORR of 19.3%, regardless of PD-L1 expression. The median duration of response was not reached and pembrolizumab was well-tolerated [20]. Based on these data, the US Food and Drug Administration (FDA) granted approval of both nivolumab and pembrolizumab for patients with ES-SCLC with disease progression on or after platinum-based chemotherapy and at least one other prior line of therapy. Unfortunately, nivolumab monotherapy later failed in demonstrating OS advantage in the phase 3 trial Checkmate-331 [21].

Moving to anti-PD-L1 agents, atezolizumab monotherapy showed inconsistent results in phase 1a and phase 2 trials [22,24], while durvalumab demonstrated durable clinical activity in certain patients with pretreated ES-SCLC in a phase 1/2 trial [25].

Durvalumab has also shown clinical activity with a manageable safety profile in combination with the anti-CTLA-4 agent tremelimumab in pretreated ES-SCLC. In a phase 1 study, the confirmed ORR was 13.3% and the median PFS (mPFS) and mOS were 1.8 months and 7.9 months, respectively. Responses were durable and were observed in both platinum-sensitive and platinum resistant/refractory patients [26]. These results were confirmed in Arm A of BALTIC, a phase 2 multi-arm trial, in which durvalumab and tremelimumab in platinum-refractory or resistant ES-SCLC showed similar results with an ORR of 9.5% and an mOS of 6 months, with a tolerable safety profile [29].

The phase 2 ongoing BIOLUMA trial (NCT03083691) includes two cohorts of patients with lung cancer (non-squamous NSCLC and SCLC). In the SCLC cohort, after progression to platinum-based first-line therapy, patients receive four cycles of nivolumab (1 mg/kg) in combination with ipilimumab (3 mg/kg) and subsequent nivolumab monotherapy (240 mg flat dose). In the initial all-comers SCLC cohort, a 38% ORR (primary endpoint) had been reported [30], but the protocol was then amended to include patients with high TMB only, with the aim to understand the role of TMB as a prognostic response factor to ICIs, and it is currently ongoing [31].

If overall anti-PD-(L)1 agents provided signals of efficacy in relapse/refractory SCLC, the maintenance strategy after first line chemotherapy does not seem to improve patients’ outcome over observation. Indeed, neither the combination of nivolumab plus ipilimumab nor pembrolizumab alone prolonged survival compared with placebo in phase 3 and phase 2 studies, respectively [27,28].

After these preliminary results of anti-PD-(L)1 monotherapy, and given the premise that combining chemotherapy with immunotherapy might further enhance tumor antigenicity, ES-SCLC has also been hit by the current wave of combination strategies, leading to a substantial change in the first-line setting.

Before shifting to first line treatment, a combination strategy has also been explored in the second line. In a phase 2 trial, patients with ES-SCLC who had a disease progression after first-line EP were treated with paclitaxel (175 mg/m^2^ every 3 weeks) for six cycles, with the addition of pembrolizumab (200 mg) from the second cycle until progression or unacceptable toxicity. The results were encouraging, as a 23.1% ORR (primary endpoint) was reported, with mPFS and mOS of 5.0 and 9.1 months, respectively. Given these results, the authors concluded that pembrolizumab plus paclitaxel showed a moderate activity with acceptable toxicity in patients with refractory ES-SCLC [23].

Given the aggressiveness and the tendency to a rapid progression of disease, the combined approach of chemotherapy and immunotherapy became the preferred upfront treatment. This strategy allows for the treatment of a wider number of patients with immunotherapy and to treat them while they are still reasonably fit, as the clinical behavior of SCLC progressively reduces the number of patients who are able to receive second line treatments.

## 3. The Establishment of a New First-Line Standard

The first monoclonal antibody investigated in combination with chemotherapy for the treatment of ES-SCLC was ipilimumab [32], which actually failed in demonstrating OS advantage when combined with EP in a phase 3 trial [12].

In the last years, new standards of care were established in first-line therapy of ES-SCLC based on two double-blind, phase 3 randomized trials: Impower133 and CASPIAN [13,15].

In the Impower133 trial, 403 patients were randomly assigned to receive EP with either atezolizumab or placebo (four cycles), followed by a maintenance phase with atezolizumab/placebo until unacceptable toxicity, disease progression or loss of clinical benefit. The study met both its primary endpoints. Indeed, at a median follow-up of 13.9 months, the addition of atezolizumab to chemotherapy significantly prolonged OS (12.3 vs. 10.3 months; HR 0.70 [95% CI 0.54–0.91]; *p* = 0.007) and PFS (5.2 vs. 4.3 months; HR 0.77 [95% CI 0.62–0.96]; *p* = 0.02) [13].

With a similar design, the CASPIAN trial assessed durvalumab, with or without tremelimumab, in combination with etoposide plus either cisplatin or carboplatin in treatment-naive patients with ES-SCLC. Patients were randomly assigned to durvalumab plus EP, durvalumab plus tremelimumab plus EP, or to chemotherapy alone. Although no substantial differences were found in terms of PFS, the addition of durvalumab to first line chemotherapy significantly improved OS in patients with ES-SCLC (12.9 months vs. 10.5 months; HR 0.75 [95% CI 0.62–0.91]; *p* = 0.0032) [33]. On the other hand, durvalumab plus tremelimumab plus EP was not associated with significant improvement in OS when compared with EP despite a numerical improvement being seen after 3-year follow-up [16,33].

Indeed, in both trials the advantage of a combination strategy has been confirmed with a longer follow-up [14,16], and safety findings were consistent with the known safety profiles of all drugs administered [13,15].

Based on these results, despite non impressive benefits, in March 2019 the FDA (and followed shortly after by the European Medicines Agency (EMA)) approved atezolizumab in combination with EP for the first-line treatment of patients with ES-SCLC. A year later, even durvalumab combined with a platinum-based regimen received FDA approval in the same setting.

The phase 3 study Keynote-604 also supports the benefits of pembrolizumab in untreated ES-SCLC [17]. The study compared pembrolizumab plus EP vs. placebo plus EP. PFS was significantly prolonged by the addition of pembrolizumab (HR 0.75 [95% CI 0.61–0.91]; *p* = 0.0023), while the significance threshold for OS was not reached, probably also due to the statistical plan of the study. Regardless, a trend toward improved OS with pembrolizumab was observed. A benefit with pembrolizumab was, however, reported in all subgroups, except among patients with brain metastases (that were also more represented in this trial than in CASPIAN or Impower133), but this was not enough to place the experimental strategy in current clinical practice [17]. Very recently, at the IASCL World Conference on Lung Cancer 2022, updated survival results from this trial were presented, reporting median OS of 10.8 and 9.7 months in the immunotherapy and chemotherapy arm, respectively (HR 0.76). The authors also underlined that 18 patients completed all 35 cycles of pembrolizumab, and that 72.2% of them were still alive 2 years after the end of pembrolizumab treatment, suggesting that some patients may derive great benefit from treatment.

Furthermore, a Chinese phase 3 trial of first line chemo-immunotherapy was recently published [19]. The experimental treatment consisted of a combination of carboplatin and etoposide with adebrelimab (a novel anti-PD-L1 agent). Chemotherapy was administered together with adebrelimab for four to six cycles, followed by adebrelimab maintenance. OS was significantly improved by adebrelimab (mOS 15.3 vs. 12.8 months [HR 0.72 95% CI 0.58–0.90]; *p* = 0.0017), with an acceptable safety profile [19]. Notably, hematological adverse events were the most common in both groups, suggesting that they were mainly related to chemotherapy. In particular, grade 3–4 neutropenia was reported in 76% of patients, while anemia and platelet count decrease were mainly of grade 1–2 (57% and 45%, respectively). With the limitations of comparison among different studies, hematological toxicity rates were higher than those reported in the CASPIAN and Impower133 trials, probably due to the possibility of administering more cycles of chemotherapy in the Capstone-1 study (median number of cycles was six in both arms) [19].

Tislelizumab, an anti-PD-1 antibody with high affinity and specificity, was recently tested in Chinese patients in a phase 2 study in combination with platinum-based chemotherapy as the first-line treatment for advanced NSCLC (both squamous and non-squamous) or SCLC. Specifically, patients with ES-SCLC were treated with EP + tislelizumab 200 mg for four to six cycles, and reported 77% ORR (primary endpoint). The SCLC cohort was characterized by high cell cycle and DNA repair gene expression, and lower expression of inflammatory and immune-related genes. Moreover, PD-L1 expression on tumor cells did not correlate with the efficacy of the combination treatment [18].

Further studies are ongoing to consolidate the role of ICIs as first-line treatment and in recurrent/relapsed ES-SCLC in an effort to enhance their efficacy with many strategies, including the addition of different agents such as the antiangiogenetic drug bevacizumab (NCT04730999) [34], and these are reported in Table 2.

## 4. Combining Immunotherapy and Ionizing Radiation for SCLC

The combination of radiation therapy and ICIs showed a synergistic effect in xenograft models of different cancers [51,52]. This may happen because tumor-antigen release induced by radiations can activate an immune response directed against the antigen itself [53,54]. The addition of radiotherapy to immunotherapy allows for the boosting of the immune system, stimulating the release of immunogenic tumor-specific antigens and depleting intratumoral T_regs_, thus encouraging the idea that the combination of radiotherapy with immunotherapy may strengthen the anti-tumor immune response both locally and systemically (the so-called abscopal effect) [55,56].

Furthermore, chemotherapy or radiation therapy, reducing the tumor mass (debulking effect), may create an environment more suitable for T-cell activation and thus decrease the immunosuppressive properties of cancer [57,58]. Therefore, immunotherapy may benefit from concomitant treatment with chemoradiotherapy in SCLC for an enhanced effect.

### 4.1. Limited Stage SCLC

Limited-stage small-cell lung cancer (LS-SCLC) represents a third of all cases of SCLC with median OS between 15 and 30 months. Current European guidelines suggest concurrent chemo-radiotherapy (cCRT) as the gold standard approach in patients with stage I-III LS-SCLC and ECOG performance status 0–1 [9]. While immunotherapy is increasingly used in the management of ES-SCLC, there has been no change in the treatment of LS-SCLC in the last years. The preferred chemotherapy regimen is cisplatin plus etoposide every 3 weeks, usually performed for four cycles [2]; if not feasible, cisplatin could be switched to carboplatin, with similar or slightly worse outcomes [59].

According to Turrisi et al. and the more recent CONVERT trial, the optimal schedule is represented by concurrent hyperfractionated twice daily (BID) thoracic radiotherapy (TRT) delivering 45 Gy/30 fractions BID within 3 weeks [60,61,62]. A new trial (NCT00632853) [63] is currently ongoing, comparing the current BID regimen with an even higher QD regimen (70 Gy/35 fractions in 7 weeks) as well as one from Denmark that is evaluating two different regimens of hyperfractionated radiation therapy (THORA trial, NCT02041845) [64].

Despite this, once daily radiotherapy (60–66 Gy in 30–33 fractions) remains the most prescribed radiotherapy fractionation in cCRT for LS-SCLC [65].

According to current guidelines, patients who respond to chemo-radiation therapy, with a good performance status and no contraindication, should be offered prophylactic cranial irradiation (PCI). After PCI, close follow-up is indicated, justified by the quick rate of recurrence of this type of cancer [9]. In selected patients responding to cCRT, close surveillance using brain magnetic resonance imaging may be proposed as an alternative to PCI, as reported by Mamesaya et al. [66].

Given the recent developments in the treatment of locally advanced NSCLC [67], there is also growing interest in consolidation immunotherapy after chemo-radiotherapy in LS-SCLC. Several clinical trials are ongoing to assess the efficacy and safety of different drugs in this setting (Table 2). The phase 3 ADRIATIC trial (NCT03703297) is evaluating the efficacy of durvalumab with or without tremelimumab as consolidation therapy for patients with LS-SCLC without disease progression after CRT [68]. Another trial utilizing immunotherapy for the consolidation treatment of LS-SCLC was the phase 2 STIMULI trial which aimed to investigate whether the use of consolidation with nivolumab and ipilimumab in LS-SCLC after chemoradiation therapy and PCI was better than the standard of care. Patient recruitment closed prematurely due to slow accrual and the statistical analyses plan was updated to address PFS as the only primary endpoint that was actually not met [69]. At time of writing, the addition of immunotherapy to standard chemoradiotherapy in LS-SCLC is being actively investigated in different ongoing studies (Table 2). NRG-LU005 (NCT03811002) is a phase 2/3 trial that is currently recruiting LS-SCLC patients to evaluate the addition of concurrent durvalumab to standard chemoradiation, with OS and PFS as primary endpoints [42].

Lastly, the ACHILES trial will investigate whether adjuvant atezolizumab treatment after standard cCRT improves survival compared with observation [40].

### 4.2. Extended Stage SCLC

After first-line chemo-immunotherapy, approximately 75% of ES-SCLC patients have residual intra-thoracic disease, and approximately 90% of patients will progress with intrathoracic disease [70]. Hence, consolidative thoracic radiation has been thoroughly investigated with the aim of improving outcomes, both in terms of disease control and survival [71,72,73,74]. The CREST trial, in particular, showed a survival benefit from consolidative TRT in patients responding to first-line chemotherapy [71]. Secondary post-hoc analysis showed an improvement in survival in patients with residual disease following systemic therapy [75] and with two or fewer extra-thoracic sites of disease [61].

Despite immunotherapy alone and TRT alone being proven to improve the outcomes of ES-SCLC patients, the combined regimen has been under investigated. Indeed, many concerns were reported about the possible development of immuno-related toxicity caused by ICIs and radiotherapy combination. However, favorable safety profile data were reported by Welsh et al., who evaluated the use of pembrolizumab in combination with thoracic radiotherapy after first line chemotherapy in ES-SCLC [76]. In this trial, concurrent pembrolizumab was given with cCRT for up to sixteen cycles and was well-tolerated, with few high-grade adverse events and no dose-limiting toxicities (DLTs). The primary endpoint for the phase 1 portion was safety, while secondary outcomes included PFS, OS, and tumor response. Reported ORR was 79%. The incidence of pneumonitis was 15% (none grade 4), with grade 3 pneumonitis reported in patients exposed to a higher dose of radiation, suggesting that these patients may need dose modifications and increased attention when they are treated with ICIs.

Furthermore, a phase 2 study was designed to investigate the safety and efficacy of combination therapy (durvalumab plus tremelimumab) with or without stereotactic body radiation therapy (SBRT) in relapsed SCLC. This study proved this regimen to be safe, but without relevant improvements in terms of PFS and OS [77].

Due to these findings, several clinical trials are currently ongoing to investigate the combination of immunotherapy and radiotherapy to exploit its synergistic effects in patients with SCLC, as well as immunotherapy maintenance after chemoradiation treatment (Table 2). Based on the concept of radiation and immunotherapy synergy in ES-SCLC, RAPTOR (NCT04402788) [50] is a phase 2/3 randomized controlled trial that will test the efficacy of consolidative thoracic radiation and SBRT to metastatic sites of disease after four cycles of platinum plus etoposide plus atezolizumab. Two studies are currently assessing the combination of immunotherapy and radiation treatment after chemotherapy, with pembrolizumab (NCT02402920) [45] and ipilimumab plus nivolumab (NCT03043599) [78], respectively. Finally, two phase 2 trials are currently exploring the recurrent-SCLC scenario: the first one will examine nivolumab plus ipilimumab as the treatment for SCLC recurrence after chemo-radiation (NCT03670056) [35], while the second study will investigate the efficacy of atezolizumab plus sequential hypofractionated radiotherapy (SHRT) (NCT03262454) [48].

A further step could be the addition of other immune-modulating molecules, one of them being poly (ADP-ribose) polymerase (PARP)-inhibitors. The rationale is based on the finding that PARP- inhibitors seem to be radiosensitizers and immune modulators in preclinical settings [79,80]. PARP-inhibitors are under evaluation in SCLC in many settings and trials: among others, NCT04624204 is a double-blind phase 3 trial that aims to evaluate PFS and OS of cCRT plus pembrolizumab followed by pembrolizumab consolidation ± olaparib in LS-SCLC [43].

Ultimately, the possibility of selecting those patients who are likely to respond to different immunomodulating and combination strategies will be crucial for their success and consequently for improvements in outcomes and quality of life for SCLC patients. Thus, finding biomarkers predictive of response to therapy and a prolonged detailed follow-up are an absolute need, and will be further investigated in future studies.

## 5. Chasing Predictive Biomarkers for SCLC Immunotherapy

Although ICIs can provide substantial clinical benefit in some of patients with SCLC, there is still a relevant percentage of patients that do not respond to immunotherapy [13,15]. There is, therefore, the need to identify predictive biomarkers of response in SCLC in order to assign the right patient to the right treatment, but in randomized trials biomarkers such as smoking habits, TMB and PD-L1 expression failed in predicting patients’ outcome, and identifying patients with greater benefit [13,15,17].

Unlike for NSCLC, PD-L1 expression is indeed typically low or absent in SCLC, and its use as a predictive biomarker is limited [81]. In phase 3 trials of chemotherapy plus PD-(L)1 checkpoint inhibitors, a survival benefit was observed across different PD-L1 subgroups, and thus in clinical practice ICIs can be administered for ES-SCLC regardless of PD-L1 status [13,15,17,19].

The CheckMate-032 trial did not support the use of PD-L1 as a biomarker in SCLC: in the examined patients, PD-L1 expression was rare (only 17%), and clinical benefits were independent of PD-L1 expression. On the other side, 211 out of the 245 patients enrolled whole exome sequencing (WES) on tissue samples was performed, and survival analysis by TMB was evaluated. Patients with high TMB (defined as ≥248 mutations) experienced enhanced efficacy with immunotherapy compared to those with low (<143 mutations) or medium TMB (143–247 mutations), especially with the combination of nivolumab plus ipilimumab [82]. Furthermore, in the maintenance trial CheckMate-451, despite the lack of OS advantage in the overall population, OS improvement with both combination and nivolumab monotherapy was described in patients with higher TMB (defined as ≥13 mut/Mb), while a cutoff of 10 mut/Mb did not show any predictivity for OS [27].

The predictive role of TMB assessed by next generation sequencing (NGS) was explored in a retrospective study that used targeted sequencing data to assess the impact of TMB on ICIs efficacy (anti PD-1 and/or anti-CTLA4) in a cohort of patients with relapsed/refractory SCLC. On 52 patients, it was seen that patients with “TMB high” (defined as TMB above the 50th percentile) had significantly longer mOS and mPFS than those with “TMB low” when treated with immunotherapy [83].

On the other hand, in the updated analysis of the Impower133 trial, a survival benefit was observed regardless of blood-based TMB (with both cutoffs of 10 and 16) [14], thus questioning its possible predictive value for treatment naïve patients. This was actually an exploratory analysis that does not allow for clear conclusions.

Limitations of TMB as biomarkers include the lack of shared diagnostic methods and cutoff values. Furthermore, in SCLC diagnostic assays and biomarkers, evaluation is often limited by the inadequacy of biopsy samples. This has led also to the search for possible surrogate hematologic parameters, such as neutrophil-to-lymphocyte ratio (NLR), platelet-to-lymphocyte ratio (PLR), and systemic immune-inflammation index (SII), which reflect the balance between inflammation and immunoreaction and have already been shown to be useful in predicting outcomes in patients with melanoma and NSCLC treated with immunotherapy [84,85].

A retrospective study evaluated whether these parameters could predict response to treatment in patients with ES-SCLC treated with immunotherapy in second or subsequent lines of treatment. Forty-one patients were evaluable, and the study showed that NLR at six weeks from beginning of treatment (but not baseline NLR) related with mPFS [86].

Even changes in serum cytokine levels have been described as a possible biomarker in SCLC treated with ipilimumab [87].

In 2019, an ancillary study of the phase 2 IFCT-1603 trial was published. Its aim was to evaluate whether circulating tumor DNA (ctDNA), analyzed at the beginning of treatment, was associated with prognosis of SCLC patients treated with second line therapies (chemotherapy or atezolizumab). Patients with detectable ctDNA had significantly lower disease control at 6 weeks than patients without detectable ctDNA, regardless of the nature of treatment (29.5% vs. 58.8%, respectively; *p* = 0.030). However, it was interesting to note that the benefit in OS associated with low ctDNA was more pronounced in patients treated with atezolizumab than in patients receiving conventional second line chemotherapy. The most frequent mutations were TP53 (65.3%) and Rb1 (51.0%). Patients treated with immunotherapy had a significantly lower disease control rate when a circulating mutation was detected (13.3% vs. 50%; *p* = 0.0145), while this difference did not emerge among patients treated with chemotherapy (64.3% vs. 71.4%; *p* = 0.672). Chemotherapy appeared to be more effective than atezolizumab in patients with higher baseline ctDNA levels, while immunotherapy tended to be more effective than chemotherapy in patients with low ctDNA concentrations at baseline. These preliminary results suggest that the prospective evaluation of ctDNA in SCLC may be useful [88].

Evidence about the role of the tumor microenvironment (TME) in SCLC is limited and mainly retrospective [89,90,91,92,93], thus suggesting that a higher concentration of tumor infiltrating lymphocytes (TILs) may be related with better prognosis, even if experiences on TME and immunotherapy outcome are lacking. However, preclinical experiences are suggesting that the targeting of proteins expressed on immune cells that comprise the tumor microenvironment (e.g., CD47 or CD56) may become a therapeutic option in the future [94,95,96,97].

## 6. Future Immunotherapeutic Perspectives for SCLC

**Figure 1:**
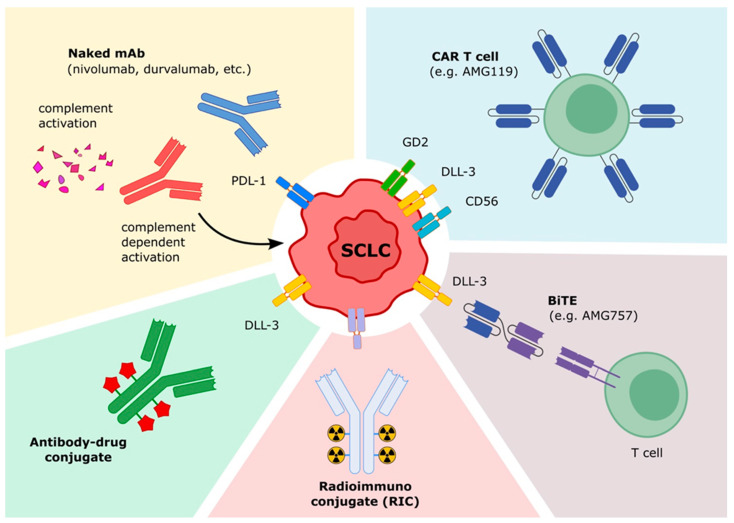
Novel immunotherapeutic strategies against SCLC. Naked mAbs act against SCLC with a different mechanism of action including the inhibition of the interaction between ligand and receptor, direct cytotoxicity of the antibody, induction of complement dependent cytotoxicity and by antibody-dependent cellular toxicity. CAR T cells. Genetically modified T cells are engineered to express a Chimeric Antigen Receptor to target a TAA. The binding of the CAR with the antigen triggers the activation of the CAR T cell which releases perforin, granzyme and other cytokines with a cytotoxic effect. Bispecific T cell Engager (BiTE). BiTEs are composed by two ScFV moiety bound together. One recognized the TAA and the other one identified the CD3 of the T cell to steer the immune response directly to SCLC. Radioimmunoconjugate (RIC). RICs are antibodies linked to a radioactive substance that might be exploited to treat SCLC, taking advantage of the specificity of a mAb and the cytotoxic effect of a radioactive compound. Antibody-Drug conjugate (ADC). ADCs are cytotoxic molecules or proteins bound to an antibody directed against a TAA (e.g., Rova-T) to more efficiently target the tumor.

Recent advances in treating SCLC shed light on the pivotal role of the immune system. In addition to checkpoint inhibitors, immunotherapy offers several different strategies, including naked monoclonal antibodies (mAbs) targeting a TAA, conjugated antibody, bispecific antibodies, immunocytokines, radioimmunoconjugate antibodies and cellular therapies (Figure 1) [98].

### 6.1. Antibody-Drug Conjugates (ADCs)

Antibody-drug conjugates (ADCs) are recombinant antibodies bound to a chemotherapeutic compound by a synthetic linker. Such an approach combines the targeting precision of an antibody with the cytotoxic activity of small molecules, consequently reducing the treatment side effects. Different generations of ADCs have been developed over the last decades, and many chemical modifications have been performed to enhance their activity. Linker, antibody modifications and different payloads have led to three different ADC generations. Immunoconjugates have several proposed mechanisms of action: release of the payload within the tumor microenvironment, the antibody engagement between cytotoxic immune cells (e.g., Fc-mediated stimulation of NK cells), and disruption of cell signaling upon antibody binding with the receptor. ADCs might be internalized by antigen-dependent pathways and the conjugated compound might be released from endosomes or lysosomes inside the cell and membrane-permeable payloads might carry out a bystander effect entering the cells nearby [99,100]. Several ADCs have been designed to target the Delta-Like Ligand 3 (DLL-3), an inhibitor of the Notch pathway highly expressed in SCLC. Rovalpituzumab tesirine (Rova-T) is an IgG1 which binds DLL-3 and it has been conjugated with the cytotoxic pyrrolobenzodiazepine (PDB) dimer [101,102]. Rova-T has been evaluated in different settings and schedules (alone or in combination with platinum-based chemotherapy or immunotherapy) for the treatment of SCLC, however, many toxicities or efficacy issues were highlighted, and its development was discontinued [103,104,105,106,107]. In particular, Rova-T monotherapy failed in demonstrating its effectiveness as a second line treatment (compared with topotecan) in a phase 3 trial and as a maintenance treatment after first-line chemotherapy (compared with placebo) [104,106]. On the other side, it demonstrated promising results in combination with budigalimab (anti-PD-1 antibody) in the expansion arm of a first-in-human phase 1 trial (NCT03000257) [108]. The development of budigalimab is ongoing in different solid tumors including NSCLC and SCLC (NCT03639194) [109].

### 6.2. Radioimmunoconjugate

Radioimmunoconjugates are antibodies linked to a radionucleotide or a radioactive compound. Different radioimmunoconjugates have been tested against blood malignancies and some solid tumors, but none was engineered to target SCLC as far as we are aware. Research on this immunotherapeutic approach has been hampered by the toxicity manifestations and the lack of results on solid tumors. However, Ibritumomab tiuxetan for non-Hodgkin Lymphoma patients is an anti-CD20 mAb conjugated with tiuxetan, a linker molecule that permits the delivery of Yttrium-90 on the tumor site. The administration of the radioimmunoconjugate produced a 74% overall response rate, with 15% of patients achieving a complete response. The median time to progression (TTP) reported was 6.8 months and extended up to 8.7 months in patients that showed a response to treatment [110]. To date, data on solid tumors are scarce. Best results have been obtained on tumors with high antigen expression, but outstanding successes are still missing. The lack of significant efficacy is based on different reasons such as poor perfusion and heterogeneous blood flow, the presence of tumor stroma and heterogeneity in targeted antigen expression. Thus, targeting patients with minimal disease burden, using pre-targeting strategies, new therapeutic radionucleotides, using combinatorial treatments and locoregional administration are the solutions proposed to overcome the hurdles towards a successful treatment. At the moment, several phase I/II clinical trials have been carried out and are still ongoing. Specifically, radioimmunoconjugates have been tested on renal cell carcinoma, prostate cancer, colon cancer, ovarian cancer, pancreatic ductal adenocarcinoma (PDAC) cancer and primary brain tumor. Therefore, the conjugation of a radioactive compound to an antibody which precisely targets SCLC would represent a novel therapeutic option for this disease, broadening clinicians’ array of therapeutic solutions [111].

### 6.3. Bispecific T-Cell Engager (BiTEs^®^)

Bispecific T-cell Engager (BiTEs^®^) are bispecific antibodies (bsAbs) composed of two different single-chain fragment variables (ScFVs) linked together [112,113,114]. Normally, bsAbs simultaneously bind two different antigens on cancer treatment, but when it comes to BiTEs one of the targets of the two ScFVs is required to engage T cells. Therefore, they are usually engineered to bind CD3 for T cell recruitment and a TAA to direct the immune response against cancer. Given their biochemical structure, BiTEs are considered proteins of small molecular size. Their chemo-physical features contribute to their swift distribution after the infusion and their high precision in targeting the tumor cell of interest [115].

AMG 757 is a BiTE^®^ that bears an ScFv binding DLL-3 and an ScFv which targets CD3e. CD3 ScFV engages T cells and the DLL-3 ScFv elicits the immune response specifically towards SCLC. AMG 757 showed efficacy in vivo on immunocompromised NOD SCID gamma (NSG™) mice and based on those results, a phase 1, open-label, multiple-dose trial to assess the safety and tolerability profile of AMG757 is ongoing (NCT03319940) [116,117].

### 6.4. Bispecific Antibody

Bispecific antibodies might be divided into bsAbs with Fc domains and bsAbs without Fc domains. The Fc domain contributes to the maintenance of the stability, simplification of the purification process, an extended half-life and the capability to be recognized by the Fc receptors on Natural Killer (NK) cells, monocytes and macrophages. The latter are smaller compounds usually composed of the binding moieties of two different antibodies (e.g., BiTEs). Bispecific antibodies due to their capability to target two different TAAs increase the treatment specificity and efficacy. Furthermore, they represent a valid solution in tackling the antigen escape phenomenon for which a tumor stops expressing a TAA targeted by a specific antibody on its surface [115]. An Open-label, Single-arm, Multicenter, Phase 1/2 Trial (NCT04750239; 402) that was terminated in April 2022 due to business priorities. During the trial, Nivatrotamab an Anti-GD2 × CD3 Bispecific Antibody, was tested in relapsed/recurrent SCLC [118]. GD2 is a disialoganglioside aberrantly expressed on neuroectoedermal origin tumors with a highly restricted expression on healthy tissues [119]. Several SCLCs and NSCLCs express GD2, which makes this antigen an attractive target to develop novel strategies against [120,121].

### 6.5. Chimeric Antigen Receptor (CAR) T Cells

One of the most remarkable breakthroughs within the immunotherapy field is represented by Chimeric Antigen Receptor (CAR) T cells. CAR T cells are genetically modified T lymphocytes which express a transgenic receptor composed of an extracellular ScFv capable of binding a TAA and by an intracellular signaling domain which triggers the T cell cytolytic properties upon the antigen engagement. Both ScFV and the intracellular domain, composed by costimulatory domains, can be modified to confer precise features to the T cell, such as enhanced persistence in vivo, increased cytokine release or resistance to exhaustion. This novel cellular therapy merges the targeting precision of a mAb with the cytotoxic power of T lymphocytes, which can trigger a robust immune response against cancer cells. Initially, CAR T cells therapy was developed for blood malignancies; however, this immunotherapeutic approach has been reconsidered for solid tumors as well [122,123,124,125]. When it comes to design an immunotherapeutic strategy against solid tumors, the choice of a valid TAA plays a non-trivial role. Anti-CD56 CAR T cells have been developed against SCLC, showing promising results in pre-clinical studies on NSG mice [126]. However, CD56 is highly expressed on SCLC, but its expression is not limited to the tumor only, leading to possible on-target off-tumor CAR T cells activation, contributing to significant side effects [127]. A phase 1 study is currently ongoing to assess the tolerability and safety of the AMG 119, an anti-DLL-3 CAR T, against SCLC used in combination with the BiTE^®^ AMG757. Even if the results have not been disclosed yet, DLL-3 expression is lower in healthy tissues compared to CD56, hence we suppose the safety profile is better for the anti-DLL-3 CAR T rather than the anti-CD56 one [102]. Nevertheless, GD2 is also considered one of the most suitable targets to design a CAR T treatment against SCLC due to its limited expression in healthy tissues. Moreover, anti-GD2 CAR T cells have been developed against several diseases such as melanoma, Ewing’s sarcoma, and glioblastoma, and data about safety and tolerability have already been collected by phase 1 trials on anti-GD2 CAR T [128,129,130,131,132]. Reppel and colleagues designed an optimized anti-GD2 CAR T incorporating IL-15 to enhance the T cells’ persistence in vivo against SCLC and NSCLC. Results on orthotopic and metastatic mice models of SCLC showed the capability of the anti-GD2 CAR T to also completely control the disease upon a re-administration of SCLC cells after 50 days from the CAR T administration, demonstrating a strong persistence and cytotoxic activity in a relapse scenario as well [133].

While still in their infancy, these innovative approaches provide novel immunotherapeutic alternative strategies for patients as a stand-alone treatment or in synergistic fashion with other compounds to significantly improve SCLC prognosis.

## 7. Conclusions

In recent years, immunotherapy has dramatically modified cancer treatment across several malignancies also being investigated in SCLC. ICIs seem to improve patient survival in the ES-SCLC setting, even in combination with platinum-based chemotherapy that remains the backbone of first line treatment. Indeed, it remains difficult to get away from chemotherapy in the treatment of SCLC, despite second or third-line therapy demonstrating some benefits in pretreated patients. The addition of ICIs to standard chemotherapy may favor a rapid tumor shrinkage due to their synergistic effect. Furthermore, a combination strategy may also limit the detrimental effect of administration of steroids that are commonly used due to patients’ clinical conditions (e.g., symptomatic brain metastases, superior vena cava syndrome or paraneoplastic diseases).

Moreover, the administration of first-line immunotherapy has the advantage of allowing the treatment of patients when they are still fit enough, as the number of patients who can undergo a second line treatment is progressively reduced by the natural history of the disease.

On the other hand, the anticipation of immunotherapy to first line makes even more urgent the research on effective second-line strategies, since the modest results of topotecan may be even more negligible after chemo-immunotherapy, and one patient out of two would need at least one further line of treatment, as demonstrated in several phase 3 trials investigating EP plus immunotherapy [13,15,17,19]. In Europe, two options are available for first line treatment of ES-SCLC with similar schedules (atezolizumab and durvalumab) that have demonstrated similar efficacy and safety profiles. In this setting, the combination between ICIs and local treatment such as radiotherapy should also be more deeply investigated due to the encouraging results obtained in several phase 1–2 clinical trials. Finally, more evidence is needed on biomarkers that may help the physician drive the decision-making process. Efforts should be made to better identify those patients who may gain a concrete benefit from ICIs, with the additional aim of finding new possible therapeutic targets that may influence clinical outcomes in this setting. Therefore, patients’ participation in clinical trials should be recommended. Despite these first encouraging steps, we still have a long road ahead to improve SCLC outcomes.

## Figures and Tables

**Table 1:** Main clinical trials about immune checkpoint inhibitors in ES-SCLC as first, second or further lines of treatment and as maintenance after first-line platinum-based chemotherapy.

Setting	Trial	Phase	No. of Patients	Treatment Arms	End Points	ORR	mPFS Months (95% CI)	mOS Months (95% CI)
1st line	Reck M et al. JCO 2016 [12]	3	1132	EP + ipilimumab EP + placebo	OS (primary) PFS (secondary)	62% ipilimumab 62% placebo	4.6 (4.50–4.99) ipilimumab 4.4 (4.73–4.63) placebo HR 0.85 (95% CI 0.75–0.97); *p* = 0.0161	11.0 (10.45–11.33) ipilimumab 10.9 (10.02–11.50) placebo HR 0.94 (95% CI 0.81–1.09); *p* = 0.3775
	Impower133 Horn et al. NEJM 2018 [13] Liu et al. JCO 2020 [14]	3	403	EP + atezolizumab EP + placebo	OS, PFS (primary) ORR, DoR (secondary)	60.2% atezolizumab 64.4% placebo	5.2 (4.4–5.6) atezolizumab 4.3 (4.2–4.5) placebo HR 0.77 (95% CI 0.62–0.96); *p* = 0.02	12.3 atezolizumab 10.3 placebo HR 0.76 (95% CI 0.60–0.95); *p* = 0.0154 *
	Caspian Paz-Ares et al. Lancet 2019 [15] Pas-Arez et al. Ann Oncol 2022 [16]	3	805	EP + durvalumab EP + duvalumab + tremelimumab EP alone	OS (primary) PFS, ORR, Safety (secondary)	68% durvalumab 58% EP alone	5.1 (4.7–6.2) durvalumab 5.4 (4.8–6.2) EP alone HR 0.78 (95% CI 0.65–0.94)	12.9 (11.3–14.7) durvalumab 10.5 (9.3–11.2) EP alone HR 0.71 (95% CI 0.60–0.86); *p* = 0.0003 *
	Keynote-604 Rudin CM et al. JCO 2020 [17]	3	453	EP + pembrolizumab EP + placebo	OS, PFS (primary) ORR, DoR, Safety (secondary)	70.6%pembrolizumab 61.8% placebo	4.5 (4,3–5,4) pembrolizumab 4.3 (4.2–4.4) placebo HR 0.75 (95% CI 0.61–0.91); *p* = 0.0023	10.8 (9.2–12.9) pembrolizumab 9.7 (8.6–10.7) placebo HR 0.80 (95% CI 0.64–0.98); *p* = 0.0164
	Wang et al. Lung Cancer 2020 [18]	2	17	EP + tislelizumab	ORR (primary) DCR, DoR, PFS, Safety (secondary)	77%	6.9 (4.9–10.09)	15.6 (11.79-NE)
	Capstone-1 Wang et al. Lancet Oncol 2022 [19]	3	462	CBDCA + eto + adebrelimab CDBCA + eto + placebo	OS (primary) PFS, ORR, DoR, DCR, 6 and 12 months-PFS, 12 and 24 moths-OS, Safety (Secondary)	70.4% adebrelimab 65.9% placebo	5.8 (5.6–6.96) adebrelimab 5.6 (5.5–5.7) placebo HR 0.67 (95% CI 0.54–0.83); *p* < 0.0001	15.3 (13.2–17.5) adebrelimab 12.8 (11.3–13.7) placebo HR 0.72 (95% CI 0.58–0.90); *p* = 0.0017
2nd line and beyond	CheckMate-032 Antonia et al. Lancet Oncol 2016 [11]	1/2	216	nivo 3 mg/kg nivo 1 mg/kg + ipi 1 mg/kg nivo 1 mg/kg + ipi 3 mg/kg nivo 3 mg/kg + ipi1 mg/kg	ORR (primary) OS, PFS, DoR, Safety (secondary)	10% nivo 3 mg/kg 33% nivo 1 mg/kg + ipi 1 mg/kg 23% nivo 1 mg/kg + ipi 3 mg/kg 19% nivo 3 mg/kg + ipi 1 mg/kg	1.4 (1.4–1.9) nivo 3 mg/kg 2.6 (1.4–4.1) nivo 1 mg/kg + ipi 3 mg/kg 1.4 (1.3–2.2) nivo 3 mg/kg + ipi 1 mg/kg	4.4 (3.0–9.3) nivo 3 mg/kg 7.7 (3.6–18.0) nivo 1 mg/kg + ipi 3 mg/kg 6.0 (95% CI 3.6–11.0) nivo 3 mg/kg + ipi 1 mg/kg
	Keynote-028/Keynote-158 Pooled analysis Chung et al. JCO 2020 [20]	1b and 2	83	pembrolizumab	ORR (primary) OS, PFS, DoR (secondary)	19.3%	2.0 (1.9–3.4)	7.7 (5.2–10.1)
	CheckMate 331 Reck M et al. Ann Oncol 2018 [21]	3	569	nivolumab chemotherapy	OS (primary)	14% nivolumab 16% chemotherapy	1.4 (1.4–1.5) nivolumab 3.8 (3.0–4.2) chemotherapy HR 1.41 (95% CI 1.18–1.69)	7.5 (5.7–9.2) nivo 8.4 (7.0–10.0) chemotherapy HR 0.86 (95 CI 0.72–1.04); *p* = 0.11
	IFCT-1603 Pujol et al. Thorac Oncol 2019 [22]	2	73	Atezolizumab chemotherapy	ORR (primary) OS, PFS (secondary)	2.3% atezolizumab 10% chemotherapy	1.4 (1.2–1.5) atezolizumab 4.3 (1.5–5.9) chemotherapy HR 2.26 (95 CI 1.30–3.93); *p* = 0.004	9.5 (3.2–14.4) atezolizumab 8.7 (4.1–12.7) chemotherapy HR 0.84 (95% CI 0.45–1.58); *p* = 0.60
	Kim et al. Lung Cancer 2019 [23]	2		paclitaxel + pembrolizumab	ORR (primary) PFS, OS, safety, biomarkers (secondary)	23.1%	5.0 (2.7–6.7)	9.1 (6.5–15.0)
	Sequist et al. Ann Oncol 2016 [24]	1	17	atezolizumab	PFS, OS, safety (primary) ORR (secondary)	6%	1.5 (1.2–2.7)	5.9 (4.3–20.1)
	Goldman et al. JCO 2018 [25]	1/2	21	durvalumab	Safety (primary) OS, PFS; ORR (secondary)	9.5%	1.5 (0.9–1.8)	4.8 (1.3–10.4)
	Cho et al. JCO 2018 [26]	1	30	durvalumab + tremelimumab	Safety, ORR (primary) OS, PFS, DoR (secondary)	13.3%	1.8 (1.0–1.9)	7.9 (3.2–15.8)
Maintenance	CheckMate 451 Owonikoko et al. JCO 2021 [27]	3	834	Nivo 1 mg/kg + ipi 3 mg/kg followed by nivo 240 mg (combination) nivolumab 240 mg placebo	OS (primary) PFS, ORR, DoR (secondary)	9.1% combination 11.5% nivolumab 4.2% placebo	1.7 (1.5–2.6) combination 1.9 (1.6–2.6) nivolumab 1.4 (1.4–1.5) placebo HR 0.72 (95% CI 0.60–0.87) combination vs. placebo	9.2 (8.2–10.2) combination 10.4 (9.5–12.1) nivolumab, 9.6 (8.2–11.0) placebo HR 0.92 (95% CI 0.75–1.12); *p* = 0.37
	Gadgeel et al. JTO 2018 [28]	2	45	pembrolizumab	PFS (primary) OS, RR (secondary)	11.1%	1.4 (1.3–2.8)	9.6 (7.0–12)

* Updated OS analysis. ORR: objective response rate; (m)PFS: (median) progression free survival; (m)OS: (median) overall survival; CI: confidence interval; HR: hazard ratio; DoR: duration of response; EP: platinum plus etoposide; DCR: disease control rate; CBDCA: carboplatin; eto: etoposide; nivo: nivolumab; ipi: ipilimumab; RR: response rate.

**Table 2:** Main ongoing clinical trials about immunotherapy combination/single agents and combinations with radiotherapy.

	Trial ID	Phase	Setting	Treatment Arms	Primary Endpoint(s)	Secondary Endpoint(s) *
Sistemic Treatements	NCT04730999 (CeLEBrATE) [34]	2	1st line	CBDCA + etoposide + atezolimab + bevacizumab	OS	Safety and tolerability; ORR; PFS
				→ atezolizumab + bevacizumab		
	NCT03083691 (BIOLUMA) [30]	2	Relapsed/	nivolumab + ipilimumab → nivolumab	ORR	OS; PFS; DoR
			recurrent			
	NCT03670056 [35]	2	Recurrent	nivolumab + ipilimumab → nivolumab	changes in T_eff_/T_reg_	RR; DoR; PFS
					cells ratio	
	NCT03406715 (MCC-19163) [36]	2	Recurrent	nivolumab + ipilimumab + Ad.p53-DC → nivolumab	DCR	PFS; OS; IR
	NCT02963090 (AFT-17) [37]	2	Progressed/	pembrolizumab	PFS	-
			relapsed	topotecan		
	NCT02489903 (QUADRUPLE THREAT) [38]	2	≥3rd line or 2nd platinum refractory/resistant	RRx-001 → platinum rechallenge at progression (CBDCA/CDDP + etoposide) EP alone	OS	ORR; DCR; PFS
	NCT02937818 (BALTIC) [29]	2	Platinum refractory	durvalumab + tremelimumab (Arm A)	ORR	DoR, 12-weeks DC, TTR
				AZD1775 + CBDCA (Arm B)		
				AZD6739 + olaparib (Arb C)		
Radiotherapy and Immunotherapy	NCT03585998 [39]	2	LS-SCLC	CRT + durvalumab → durvalumab consolidation	PFS	OS; Safety
	NCT03540420 (ACHILES) [40]	2	LS-SCLC after CRT (EP)	Atezolizumab	OS	PFS, best response,
				observation		AEs
	NCT04189094 [41]	2	LS-SCLC	CRT ± concurrent sintilimab	PFS	OS; ORR
	NCT03811002 (NRG LU005) [42]	2/3	LS-SCLC	CRT ± concurrent atezolizumab	OS	PFS; AEs; ORR
	NCT04624204 [43]	3	LS-SCLC	CRT ± concurrent pembolizumab → pembrolizumab ± olaparib	PFS; OS	AEs; AEs-related discontinuations; OR
	NCT03703297 (ADRIATIC) [44]	3	LS-SCLC	durvalumab durvalumab + tremelimumab Placebo	PFS; OS (durvalumab vs. placebo)	OS; ORR; PFS (durvalumab + tremelimumab)
	NCT02402920 [45]	1	LS- and ES-SCLC	CRT/RT + concurrent pembrolizumab	MTD	RR, PFS, OS
	NCT03509012 (CLOVER) [46]	1	LS- and ES-SCLC	CRT + durvalumab ± tremelimumab	DLTs/Aes	PFS; OS; ORR
	NCT03043599 [47]	1/2	ES-SCLC	Thoracic RT + ipilimumab + nivolumab	IT dose (phase I);	OS
					PFS(phase II)	
	NCT03262454 [48]	2	ES SCLC recurrent/refractory	Atezolizumab + SHRT	OS	PFS
	NCT03923270 [49]	1	ES SCLC after 1st line EP	Thoracic RT + durvalumab Thoracic RT + durvalumab + tremelimumab (75 mg) Thoracic RT + durvalumab + olaparib Thoraci RT + durvalumab + tremelimumab (300 mg)	SAEs (Phase 1); PFS (Phase 1b)	PFS; OS
	NCT04402788 (RAPTOR) [50]	2/3	consolidation after standard 1st line	RT + atezolizumab	PFS	AEs; PFS (Phase 3); PFS according to the number of visible tumors
					(Phase 2)	
				atezolizumab	OS (Phase 3)	

* The first three endpoints listed on Clinicaltrials.gov are reported. CBDCA: carboplatin; OS: overall survival; ORR: objective response rate; PFS: progression free survival; DoR: duration of response; RR: response rate; Ad.p53-DC: Dendritic Cell based p53 Vaccine; IR: immune response; DCR: disease control rate; CDDP: cisplatin; EP: platinum-etoposide; DC: disease control; TTR: time to response; AEs: adverse events; CRT: chemo-radiotherapy; RT: radiotherapy; MTD: maximum tolerated dose; OR: objective response; DLT: dose limiting toxicities; BORR: best overall response rate; SHRT: sequential hypofractionated radiotherapy.
